# COVID-19 in Northeast Bosnia and Herzegovina and patient’s length of hospitalization

**DOI:** 10.1186/s12879-021-06034-6

**Published:** 2021-04-19

**Authors:** Alma Trnacevic, Amer Mujkanovic, Noura Al-Salloum, Amra Sakusic, Emir Trnacevic, Emir Jusufovic, Fatima Hukic, Rahima Jahic, Richard Stratton

**Affiliations:** 1 Department of Infectious Diseases, Tuzla University Clinical Center, Ibre Pasica bb, 7500 Tuzla, Bosnia and Herzegovina; 2 Department for Family Medicine, King Fahad Medical City, As Sulimaniyah, Riyadh, 12231 Saudi Arabia; 3 Mayo Clinic Florida; Mid Cheshire Hospitals NHS Foundation Trust, Middlewich Rd, Crewe, CW1 4QJ UK; 4 Department of Medicine, Royal Free Trust, Pond St, Hampstead, London, NW3 2QG UK

**Keywords:** COVID-19, Bosnia and Herzegovina, Length of hospitalization

## Abstract

**Background:**

Since the outbreak of COVID-19 pandemic, clinical data from various parts of the world have been reported. Up till now, there has been no clinical data with regards to COVID-19 from Bosnia and Herzegovina (B&H). The aim was to report on the first cohort of patients from B&H and to analyze factors that influence COVID-19 patient’s length of hospitalization (LOH).

**Methods:**

This retrospective cohort study was conducted at Tuzla University Clinical Center (UKC), B&H. It involved 25 COVID-19 positive patients that needed hospitalisation between March 28th and April 27th 2020. The LOH was measured from the time of admission to discharge. Factors analyzed induced age, BMI, presence of known comorbidities, serum creatinine and O_2_ saturation upon admission.

**Results:**

The mean age was 52.92 ± 19.15 years and BMI 28.80 ± 4.22*.* LOH for patients with BMI < 25 was 9 ± SE2.646 days (CI 95% 3.814–14.816) vs 14.182 ± SE .937 (CI 95% 12.346–16.018 *p* < 0.05; HR 5.148 CI95% 1.217 to 21.772 *p* = 0.026) for ≥25 BMI. The mean LOH of patients with normal levels of O_2_ ≥ 95% was 11.667 ± SE1.202 (CI95% 8.261 to 13.739; *p* = 0.046), while LOH for patients with < 95% was 14.625 ± SE 1.231 CI95% 12.184 to 16.757 *p* = 0.042; HR 3.732 CI95%1.137–12.251 *p* = 0.03). Patients without known comorbidities had a mean LOH of 11.700 ± SE1.075 (CI 95% 9.592–13.808), while those with comorbidities had a mean of 14.8 ± 1.303 (CI 95% 12.247–17.353; *p* = 0.029) with HR2.552.

**Conclusion:**

LOH varied among COVID-19 patients and was prolonged when analyzed for BMI ≥25, comorbidities, elevated creatinine, and O2 saturation < 95%. Furthermore, risk factors for COVID-19 patients in B&H do not deviate from those reported in other countries.

## Background

In December 2019, a novel β-coronavirus emerged in the Wuhan, China, causing pneumonia-like illness. Later, this virus was identified as the severe acute respiratory syndrome-related coronavirus 2 (SARS-CoV2), which caused a widespread flu-like respiratory disease, and was named the Coronavirus Disease 2019 - COVID-19 [1, 2]. On March 11th, 2020, the World Health Organization declared COVID-19 as global pandemic [3].

The reported mortality varied from country to country, from 7.2% in Italy to 2.3% that has been reported in China [4]. Until April 28th, 2020 the total number of people who have been infected globally reached more than 3 million, with more than 200 thousand fatalities. On the same date, Bosnia and Herzegovina (B&H) reported 1585 individuals with COVID-19, of which 63 people had deceased [5]. The first COVID-19 positive case in B&H was documented on March 17th, while in the Tuzla Canton, Northeastern part of Bosnia and Herzegovina (population 477,000) the first registered case was on March 28th, 2020. This is comparably late for the first registered infection when taken into account other European nations, which had earlier detections of COVID-19 infection. In the period between 28th of March 2020 and 27th of April 2020 there were total of 90 patients positive for COVID-19 with 25 hospitalizations in Tuzla Canton [6]. This coincided with the near end of the flu season and was 14 days after lockdown measures were introduced in this part of B&H (social distancing measures due to COVID-19 were imposed on 14th of March); it is also worth mentioning that after 27th of April this region did not have any new reported infections for total of 44 days. Furthermore, this is the first research article that has analyzed hospitalized COVID-19 patients in B&H and assessed the length of hospitalization (LOH) of these patients as of yet and can provide insight into the nature of COVID-19 in this part of Europe [6–8].

SARS-CoV2 is an RNA virus, from the family of the coronaviruses, that were first identified in the 1960s, and since then, seven of the coronaviruses are known to infect humans [9]. Usually, coronaviruses cause mild flu-like symptoms and these viruses are transmitted when infected droplets come in contact with the mucous membranes of a susceptible human host, this can be either directly through person to person contact, or indirectly through contact with contaminated surface [10]. Conjunctival tears, saliva, urine and stool are also being considered as possible pathways of infection of COVID-19. The process of virulence with COVID-19 is initiated when SARS-CoV2 viruses latch on receptors of the Angiotensin-Converting Enzyme 2 (ACE2). Different levels of ACE2 among population groups were speculated as a reason behind the range of severity of inflammation. Individuals with COVID-19 infection, experience formation of hyaline membrane that increases the thickness of the alveolar wall, consequently reducing O_2_ exchange in the lungs [11, 12].

Association of obesity (BMI > 30) and severe clinical presentation in COVID-19 infected patients is currently being investigated. The mechanism on how obesity correlates to more severe outcome could be explained by the substantial respiratory system compromise paired with increased airway resistance, impaired gas exchange, a lower lung volume and weaker respiratory muscle strength. Furthermore, obese patients are more likely to have other comorbidities such as cardiovascular disease, insulin resistance or metabolic imbalances, that put these patients at risk severe form of COVID-19 infection. The risk of mortality in obese patients was recognized before COVID-19 in other viral infections, where in the previous influenza pandemics of H1N1 and H1N5, patients with a higher BMI had higher risk of lethal outcome [13, 14]. In a retrospective cohort from France which included 124 with COVID-19, that were admitted in the ICU, most of the patients who required invasive mechanical ventilation were obese, with a BMI above 35 (85.7%). Similarly, in Shenzhen, China, 32% from 383 patients with COVID-19 were overweight, while 10.7% were obese. Those who were obese had 2.42 higher odds of their disease progressing to severe form [15]. Moreover, a more extensive study from New York that included 4103 COVID-19 positive patients showed that BMI > 40 was a negative predictive factor for sever form of illness. In case of children, BMI-for-age can produce clear picture with regards to weight status of the patient and is used for children older than 2 years of age [16].

According to the CDC, older age groups as well as any age group with known comorbidities were found to have a higher risk of developing severe from a SARS-CoV2 infection. Comorbidities that were reported to be associated with severe form of COVID-19 were moderate to severe asthma, chronic lung conditions, diabetes, serious heart disease, kidney disease undergoing dialysis, chronic liver disease, cancer or immunocompromised individuals [17].

This report aims to analyze the first 25 patients who tested positive for COVID-19 in Northeaster part of B&H and whose clinical condition required hospitalization at Tuzla University Clinical Center, Bosnia and Herzegovina and the impact of different clinical characteristics and admission factors on the length of hospitalization (LOH). These patients were followed from admission to discharge. Further exploration of their clinical characteristics will be elaborated as a full understanding of the disease is still limited.

## Methods

Patients studied in this retrospective cohort study were admitted to the Infections Diseases Clinic, Tuzla University Clinical Center, Bosnia and Herzegovina, from 28th of March 2020 until the 27th of April 2020, when the last patient in this study was discharged form hospital. The diagnosis of COVID-19 infection was confirmed by oropharyngeal swab using reverse transcription polymerase chain reaction (RT-PCR) diagnostic test. Admission criteria was based on Modified Early Warning Score greater than 4 together with overall clinical presentation; while criteria for discharge were: afebrile for three consecutive days, improvement of respiratory symptoms, O_2_ saturation > 94%, no O_2_ support, as well as two consecutive COVID-19 RT-qPCR negative tests with at least 48 h in-between. Twenty-five patients were admitted to the hospital due to their clinical severity (*n* = 25). Data was collected from patients medical history and discharge reports, as well as from blood laboratory results that were collected upon hospitalization. Data included length of hospitalization, the eventual outcome of care, age, O_2_% saturation, Body Mass Index (BMI), creatinine, as well as any presence of known comorbidities. Ethical approval was granted from Ethical Committee of Tuzla University Clinical Center, Tuzla.

### Statistical analysis

Data is represented as mean ± standard deviation if not indicated otherwise. Kaplan-Meier survival analysis was used for analyzing the LOH, and the difference between two groups was analyzed using the log-rank; any *p* < 0.05 was reported as significant. Hazard ratio was calculated by Cox linear regression. The statistical analysis and graph creation were conducted using SPSS v.25 (IBM Corp., Armonk, NY, USA).

## Results

In total there were 90 patients who were confirmed to have Covid-19 in northeast part of B&H, with 25 (27.78%) needing hospitalization. Out of the 25 patients, 60% were male (*n* = 15) and 40% female (*n* = 10) The mortality rate in this study was 0%. In respect to presence of known comorbidities, 60% had one or more, while 40% had no none (Table 1).

**Table 1:** Clinical data of the hospitalized COVID-19 patients (*n* = 25)

	Overall	BMI^a^		Comorbidities^a^		Creatinine		O_2_%^a^	
		BMI < 25	BMI ≥ 25	Yes	No	Normal	Elevated	≤94% O_**2**_	≥95% O_**2**_
BMI	*28.80* *±4.22*	*22.67 ± 2.30*	*29.64 ± 3.71*	*26.10 ± 1.91*	*30.60±* *4.42*	*28.64 ± 4.41*	*30.00 ± 2.65*	*30.06 ± 4.13*	*25.57 ± 2.99*
Age (years)	*52.92* *±19.15*	*29.67 ± 28.1*	*56.10 ± 15.99*	*41.90 ± 9.63*	*60.27±* *20.60*	*50.59 ± 19.01*	*70.00 ± 10*	*42.57±* *20.14*	*57.29 ± 18.18*
Male/ female	*15/10*	*1/2*	*14/8*	*5/5*	*5/10*	*13/9*	*2/1*	*8/9*	*6/1*
Comorbidities (yes/no)	*15/10*	*0*	*14/8*	*10*	*15*	*12/10*	*3/0*	*12/5*	*3/4*
Creatinine (μmol/L)	*81.96±* *24.89*	*57.33 ± 15.89*	*85.32 ± 24.19*	*77.30±* *17.58*	*85.07±* *28.94*	*75.82 ± 17.66*	*127.00±* *26.46*	*86.86±* *37.74*	*80.35±* *19.46*
O_2_%	*92.19±* *3.99*	*94.40 ± 2.15*	*91.43±* *4.13*	*94.43±* *2.87*	*90.85±* *4.04*	*92.17 ± 4.38*	*89.25±* *1.34*	*90.82±* *3.70*	*95.51±* *2.53*
LOH (days)	*13.56*	*9.0*	*14.18*	*11.70*	*14.8*	*12.73*	*19.67*	*14.47*	*11.00*

Note: *Statistical difference between group is marked with the sign* (^a^)

The mean age of patients upon hospitalization was 52.92 ± 19.46, when divided by gender, the mean age of females was 51.4 ± 17.43 while the mean age for males was 53.93 ± 20.75. Seven patients in this group were 65 and older, with the eldest being 85 years of age and youngest being 2 years old. The overall LOH for all patients in days was 13.56 ± SE 0.93 (CI 95% 11.74–15.381;).

**Fig. 1:**
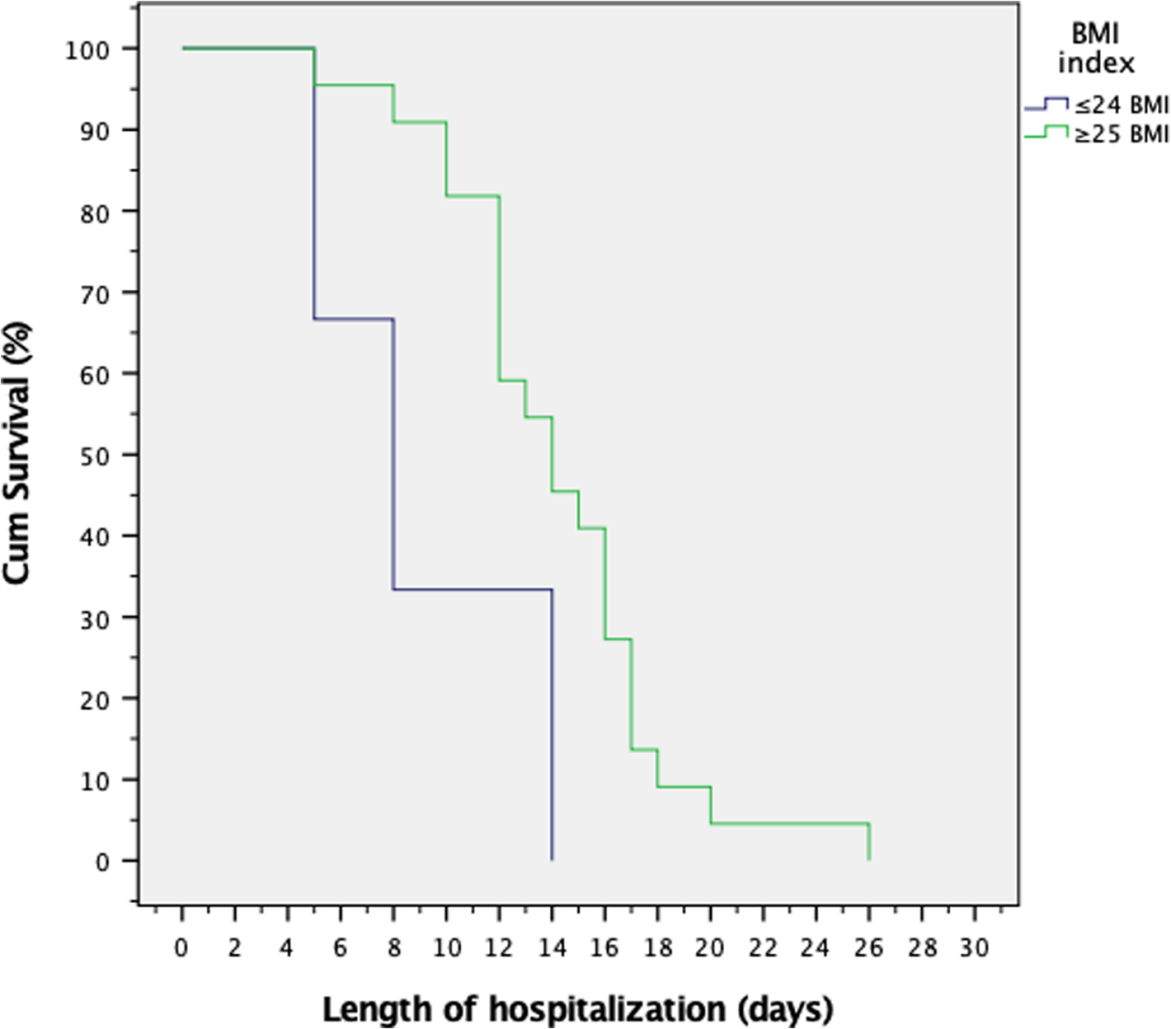
Duration of hospitalization based upon BMI. The terminal event was considered the discharge from the hospital. The blue line represents patients who have had BMI lower or equal to 24, while the green line represents the patients who had had BMI equal or larger than 25. On the 8 ^th^ day of hospitalization 95% of patient with BMI > 25 were still hospitalized while 33% in the group of patients with BMI < 25. The mean duration of hospitalization was for group 1 was 9 ± SE 2.646 (CI 95% 3.814–14.816) while for the group 2 was 14.182 ± SE .937 (CI 95% 12.346–16.018; p = 0.04)

When the mean LOH was compared to BMI the mean duration of hospitalization for patients with normal BMI was 9 ± SE 2.646 (CI 95% 3.814–14.816) while patients with BMI ≥25 had 14.182 ± SE0.937 (CI 95% 12.346–16.018; *p* = 0.040) LOH (Fig. 1). On the 8th day of hospitalization 95% of patient with BMI > 25 were still hospitalized while 33% in the group of patients with BMI < 25. Childs BMI was calculated adjusted according to criteria provided by Center for Disease Control (CDC). Using Cox regression model established HR 5.148 CI95% 1.217 to 21.772 (*p* = 0.026).

**Fig. 2:**
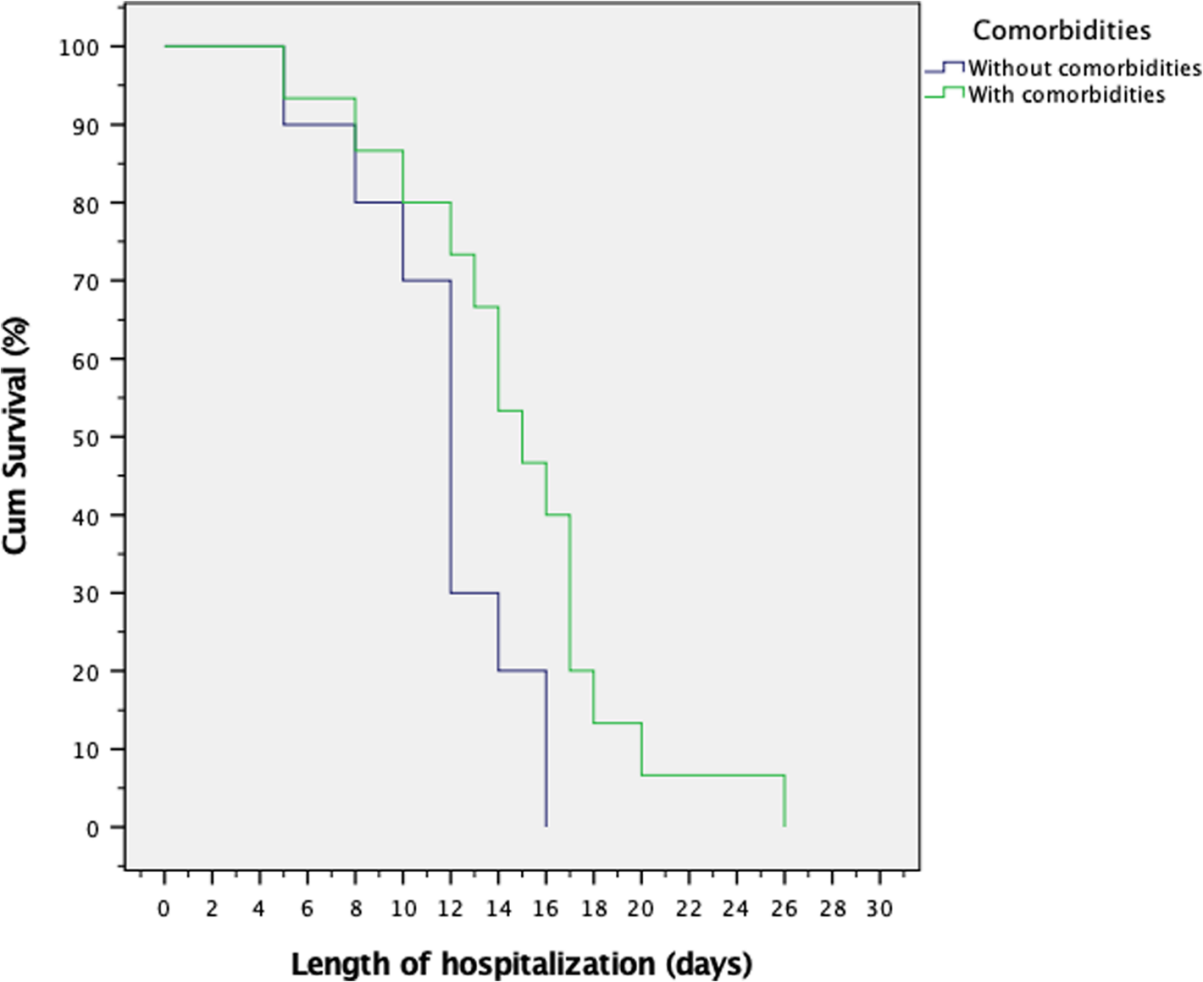
At 8th of hospitalization 80% of patients without known comorbidities were hospitalized compared to 90% of those with while on the 16th day was 0% vs 45% respectively. Duration of hospital admission based on underlying health conditions. The purple line indicates group 1 (patients without underlying diseases) had a mean duration of hospitalization 11.700 ± SE1.075 (CI 95% 9.592–13.808) while the group 2 (with comorbidities) had mean 14.8 ± SE1.303 (CI 95% 12.247–17.353; *p* = 0.029)

Patients without known comorbidities had a mean LOH 11.700 ± SE1.075 (CI 95% 9.592–13.808), while those with comorbidities had a mean of 14.8 ± 1.303 (CI 95% 12.247–17.353; *p* = 0.029). On the 8th day of hospitalization 80% of patients with known comorbidities were still in hospital whereas 85% in the group without (Fig. 2). However, on the 16th day it was 0% vs 45%, respectively. On the other hand Cox regression model did produce HR 2.552 CI95% 0.990 to 6.576 with borderline significance of *p* = 0.052.

**Fig. 3:**
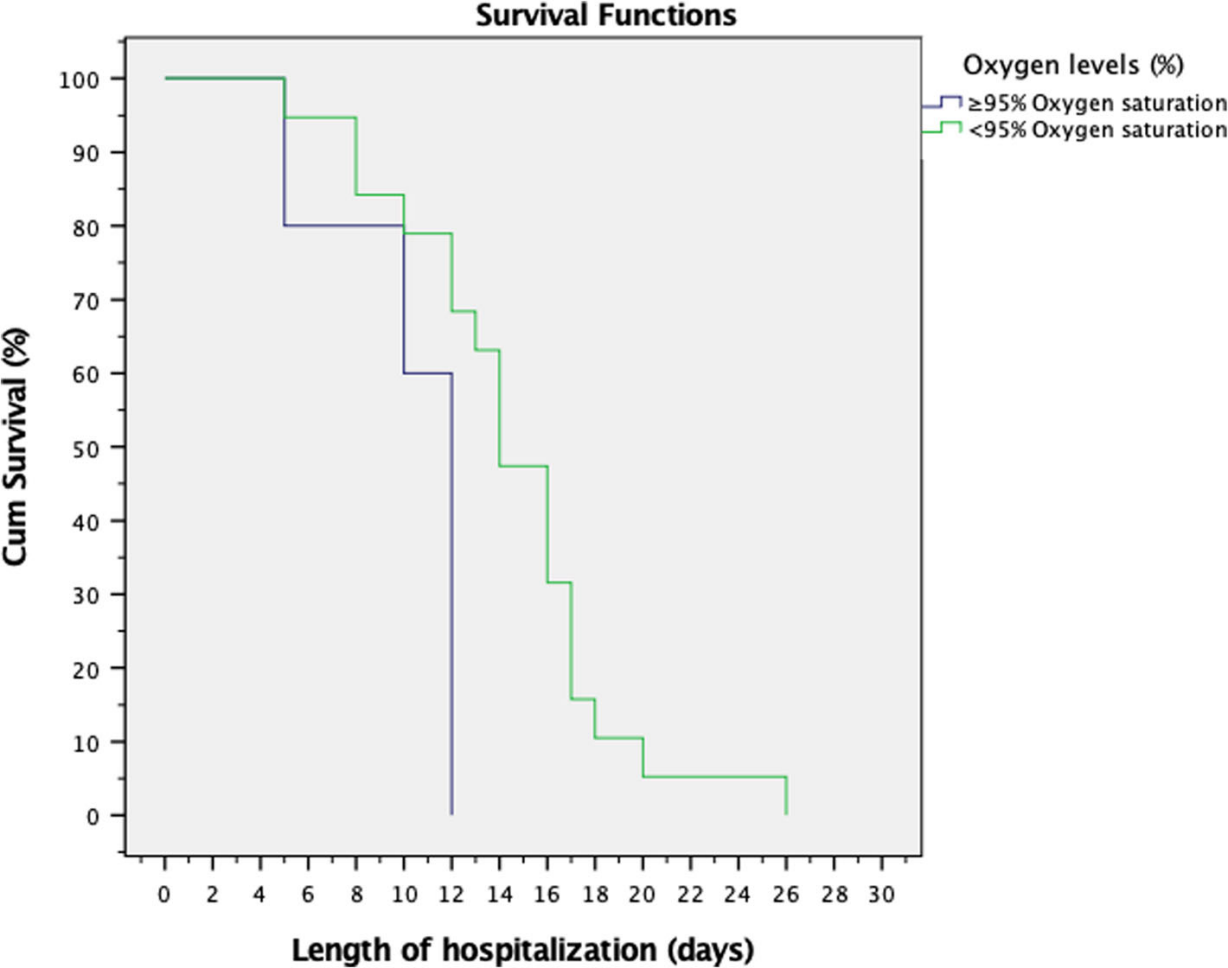
At the 8th day of hospitalization 80% of patients with normal levels of O_2_% were hospitalized compared to 84% of those with lower levels of O_2_, while at the 12th day it was 0% vs 80% respectively. Effect of oxygen saturation upon admission, on length of hospitalization of COVID-19 patients (LOH). The levels of oxygen equal or below 94% (green line) has resulted in increased duration of hospitalization 11.667 ± SE1.202 (CI 95% 9.311–14.022) while normal oxygen levels (purple line) results in decreased length of hospitalization 14.625 ± SE1.231 (CI 95% 12.212–17.038; *p* = 0.046)

The mean LOH of patients with normal levels of O_2_ ≥ 95% was 11.667 ± SE 1.202 (CI95% 8.261 to 13.739; *p* = 0.046), while LOH for patients with ≤94% was 14.625 ± SE 1.231 CI95% 12.184 to 16.757 *p* = 0.042 with HR 3.732 CI95%1.137 to 12.251 *p* = 0.03 (Fig. 3).

**Fig. 4:**
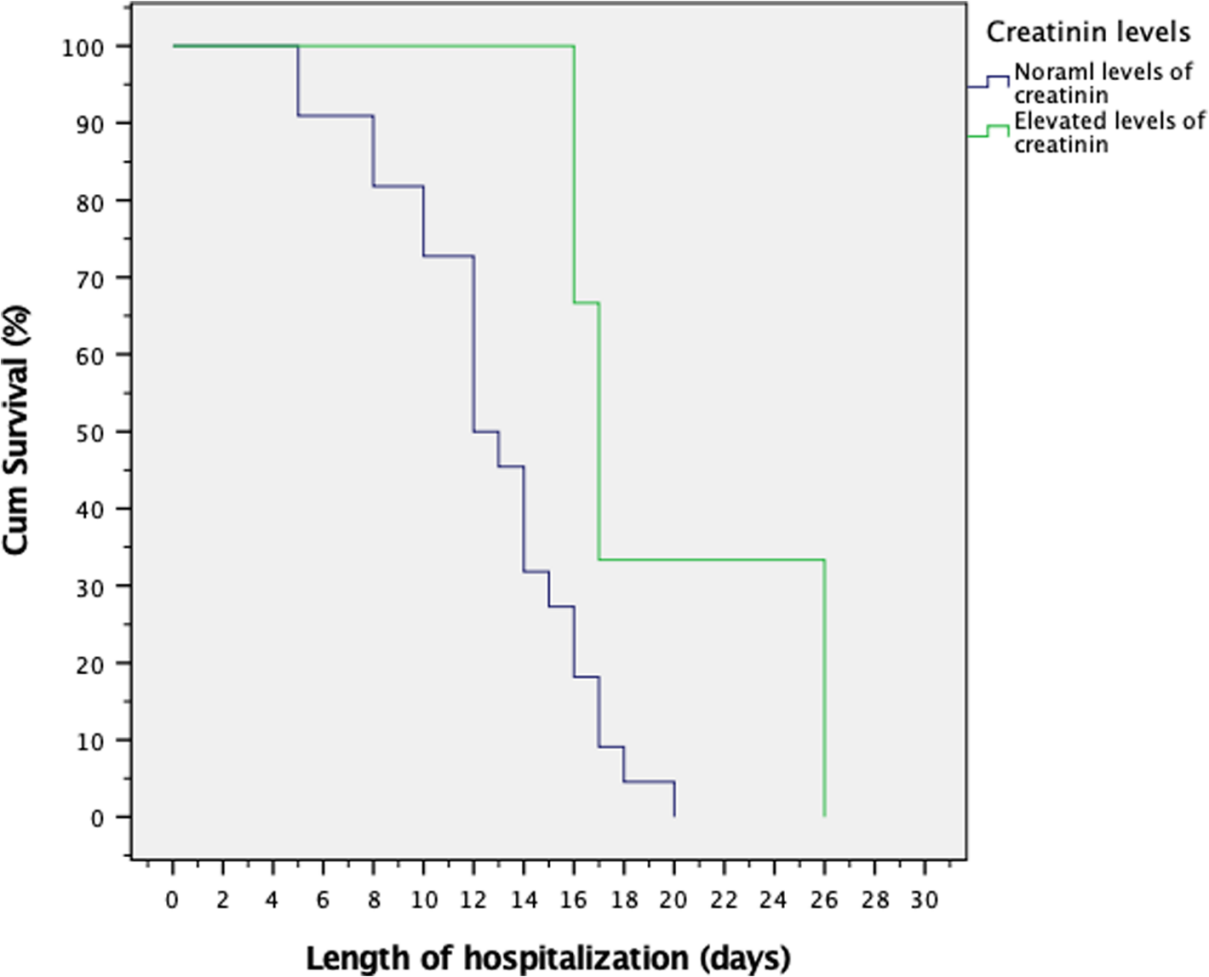
At the 8th day of hospitalization 82% of patients with normal levels of creatinine were hospitalized compared to 100% of those with elevated levels of creatinine, while at the 14th day it was 33% vs 100% respectively. Impact of elevated levels of creatinine on length of hospitalization. The patient with elevated creatinine levels (green line) had longer length of hospitalization when compared to group of patients that have had normal levels of creatinine (purple line) on admission (*p* = 0.049)

Patients with elevated creatinine levels had longer LOH 19.667 ± SE3.180 (CI95% 9.582 to 13.808; *p* = 0.049) when compared to group of patients that had a normal creatinine level upon admission 12.727 ± SE0.846 (CI95% 11.070 to 14.385; *p* < 0.05) (Fig. 4). None of these patients required dialysis during hospitalization according to KDIGO protocol [18].

## Discussion

This retrospective cohort analyzed twenty-five COVID-19 positive patients who were admitted to UKC Tuzla hospital between 28th March and 27th April, 2020. All the patients that were hospitalized in this period were included. Even though the mortality rate due to infection of COVID-19 around the world ranged of 2% up to 7.2%, the mortality rate in the region of Tuzla was 0% [4]. The possible reasons for lack of lethal cases could be explained by the younger age of patients in our study compared to the mean age in other studies; also, a high proportion of patients in this study group did not have any known comorbidity (40%) which could have had a significant effect on 0% reported mortality. Furthermore, limited testing capacity at the beginning of the pandemic could have influenced the official count of deaths due to COVID-19 and that there were deaths caused by COVID-19 but not detected. In addition, this study started in late March, which coincided with the end of the flu-season could have potentially influenced the mortality where some patients could have been misdiagnosed with influenzas instead of COVID-19. Another factor that could have contributed to low mortality is low population density of 168 /km2 of Northeast part of B&H, which is considerably lower when compared to Lombardy in Italy or New York in the United States, leading to slower spread of the COVID-19 virus.

The LOH in our cohort was affected by presence of known comorbidities, BMI > 25, elevated creatinine and blood O_2_ saturation levels. These factors could be used as predictive measures in planning patient care and may preserve hospital capacity as well as need for admission.

The effect BMI had on the LOH was significant, where patients who were categorized as overweight or obese had a longer LOH when compared to patients with a mean of 9 days for patients with BMI < 25 vs 14.18 days with BMI > 25 (*p* < 0.05) with HR 5.148. Even though our study had no mortality, it is in line with previous studies on BMI impact on disease severity and can be used as a morbidity predictor [15, 19–21]. This implies that we could possibly predict LOH and make more exact planning of hospital capacity and make more precise decision about need for hospitalization based on the BMI index of the COVID-19 patient; also, that those with BMI greater than 25 would need prolonged care and early admission to the hospital.

Patients with extensive COVID-19 associated pneumonia tend to have decreased O_2_% saturation with derangements upon admission to the hospital. COVID-19 patients exhibit impaired gas exchange across alveolar membrane with subsequent reduction in available O_2_%. In this study patients with levels of O_2_ saturation < 95% showed consistently longer LOH when compared to those with normal levels and had HR 3.732. of having prolonged LOH. Nonetheless, O_2_ levels can rapidly change in course of several hours in COVID-19 patients and thus this factor should be taken with caution and not evaluate patients solely based on this. Consequently, more dynamic and continuous evaluation of O_2_ should provide more insight into patient’s diseases severity and be supplemented with other clinical data.

In other studies, COVID-19 patients who had comorbidities on admission to hospital or critical care had higher mortality rates, as well as severe clinical manifestation. Individuals who have comorbidities are more inclined have health with reduced capacity to withstand COVID-19 infection [22]. When patients in our sample were divided into two groups, one with known comorbidities and second without known comorbidities; there was a statistical difference in the duration of hospitalization. Patients without a known comorbidity had a mean LOH of 11.700 ± SE1.075 (CI 95% 9.592–13.808) compared to 14.8 ± SE1.303 (CI 95% 12.247–17.353; *p* < 0.05) with HR of 2.552 but with borderline statistical significance. This could the consequence of smaller size of the study group and limited number of patients that were infected in the first wave of COVID-19 in the region.

Moreover, creatinine levels show the overall function of the kidney-urinary system; elevated levels could point to decrease in function or kidney injury. COVID-19 patients with an acute kidney injury in COVID-19 patients showed higher levels of hospital deaths than those with normal levels. When LOH was compared among the two groups of patients – those who had a normal level of creatinine to those with elevated – patients with elevated showed longer LOH with 19.67 ± 3.18 (CI95% 13.434–25.899) days, higher than those with normal levels 12.727 ± 0.85; CI95% 11.07–14.385; *p* < 0.05) [23].

## Conclusions

Possibility of predicting LOH of COVID-19 could be one of the crucial aspects of this pandemic, by providing additional tolls in planning hospital capacity and allocation of resources. Factors that were associated with a longer LOH was BMI ≥25 with HR 5.148, followed by O_2_ saturation levels with HR 3.732 and finally presence of known comorbidities with HR of 2.552. Despite the absence of mortality in our sample, these factors provided guidance in future planning of hospitalization of COVID-19 patients at UKC Tuzla. This is the first study regarding COVID-19 pandemic in B&H that has been written about clinical characteristics and biochemical results from COVID-19 patients. Results in this study could add to the current information about the COVID-19 pandemic in Europe. Furthermore, it provides additional information on risk factors attributed to longer hospitalization. However, this study involved a fraction of COVID-19 patients and included only a part of the B&H experience. Also, a larger sample of patients with COVID-19 is needed to make further conclusions on the nature of the pandemic in B&H. Moreover, not all biochemical and inflammatory markers that are routinely requested to monitor patients with COVID-19 were analyzed, such as d-dimer; hence, they were not included in this study. Elevated creatinine levels showed statistical difference on log-rank analyses but has not provided significant result in respect to HR, which could be verified in larger studies.

## Data Availability

Data used for this article can be found at synapse.org and synapse ID: syn22284997. Also, for any additional access to data contact the corresponding author.

## Abbreviations

ACE2: Angiotensin-Converting Enzyme 2
BMI: Body Mass Index
B&H: Bosnia and Herzegovina
COVID-19: Coronavirus Disease 2019
LOH: Length of hospitalization
RT-PCR: Reverse transcription polymerase chain reaction
SARS-CoV2: Severe acute respiratory syndrome-related coronavirus 2
